# Japanese Spotted Fever in an Elderly Patient Without Eschar and Negative Acute-Phase Serology: Diagnostic Value of Clinical Assessment and Histopathology

**DOI:** 10.7759/cureus.90645

**Published:** 2025-08-21

**Authors:** Kosuke Murashita, Natsumi Yamamoto, Shiho Amano, Kurumi Kasai, Ryuichi Ohta

**Affiliations:** 1 Community Care, Unnan City Hospital, Unnan, JPN

**Keywords:** aged, empirical therapy, general medicine, japanese spotted fever, minocycline, rickettsial infections, rural, skin manifestations, vasculitis

## Abstract

A 72-year-old woman presented with fever, generalized rash, including on the palms and soles, arthralgia, facial edema, and hepatic dysfunction. She lived in a rural area and had recent outdoor exposure to farming but no travel history. Physical examination revealed a small erythematous lesion on her right thigh, suspected to be a tick bite, though no eschar was present. Initial serological testing for *Rickettsia (R.) japonica* using the indirect immunofluorescence assay (IFA) was negative. Due to strong clinical suspicion of Japanese spotted fever, empirical treatment with oral minocycline was started, resulting in rapid resolution of fever and gradual improvement of liver function. A skin biopsy of a rash lesion revealed perivascular lymphohistiocytic infiltration and endothelial swelling, consistent with small-vessel vasculitis. Convalescent-phase serology later confirmed *R. japonica* infection with a fourfold rise in immunoglobulin G (IgG) titers. The patient fully recovered following a five-day course of minocycline. This case highlights the importance of early clinical diagnosis and empirical treatment in suspected Japanese spotted fever, especially in elderly patients with characteristic systemic symptoms and rural exposure, even when serologic confirmation is initially unavailable. Histopathological findings may support the diagnosis when serology is inconclusive. Prompt recognition and early intervention are essential to avoid complications and improve outcomes.

## Introduction

Japanese spotted fever (JSF) is a tick-borne rickettsial disease caused by *Rickettsia (R.) japonica*, typically developing several days to weeks after the bite of an infected tick [1]. The clinical spectrum can include fever, rash, lymphadenopathy, and systemic symptoms, and diagnosis often relies on a combination of epidemiological history, physical findings, and serological testing [2]. One key diagnostic clue is the detection of an eschar at the site of the tick bite. However, in older patients, these lesions may be overlooked or lost due to scratching or delayed presentation, making identification challenging [3].

Although serological tests, such as the indirect immunofluorescence assay (IFA) or enzyme-linked immunosorbent assay (ELISA), are widely used to confirm rickettsial infections, their sensitivity is limited during the early phase of the illness [4]. Antibodies typically become detectable only after 7-10 days of symptom onset, and acute-phase samples may yield false-negative results. Paired sera demonstrating a fourfold rise in antibody titers between the acute and convalescent phases are considered diagnostic; however, this delays definitive confirmation and underscores the limitation of relying solely on serology for early diagnosis. Thus, the absence of an eschar or negative serological findings cannot reliably exclude JSF, especially in the acute stage.

Here, we report the case of a 72-year-old woman who presented with fever, generalized rash, arthralgia, and facial edema after a presumed tick bite. Although her acute-phase serological test for *R. japonica *was negative, she was clinically diagnosed with JSF based on her epidemiological background and characteristic findings. She showed rapid improvement with a five-day course of minocycline.

## Case presentation

A 72-year-old woman presented with fever, generalized rash, and myalgia. She had no significant past medical history and was not taking any regular medications. She did not smoke or consume alcohol. The patient resided in the northern region of Shimane Prefecture, Japan, and regularly participated in farming activities near her home.

On day 0 (symptom onset), she developed fever, generalized erythematous rash, malaise, loss of appetite, and facial edema. On day 3, she visited the dermatology department of a rural hospital and was prescribed topical gentamicin ointment. On day 4, she consulted her primary care physician, who performed laboratory tests that revealed liver dysfunction, and she was prescribed acetaminophen 500 mg for her fever. However, her symptoms did not improve, and on day 9, she presented to the general medicine department of the rural hospital for further evaluation. She did not have any travel history, exposure to animals, or contact with infectious individuals. She did not have any other symptoms such as body weight loss, cough, chest pain, abdominal pain, back pain, night sweats, or chilly sensations.

**Figure 1:**
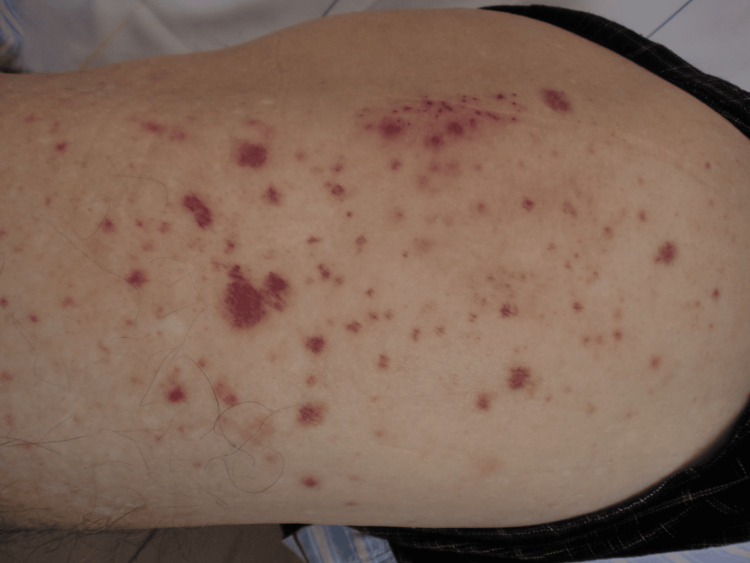
Multiple palpable purpuras diffusely observed on the lateral part of the right thigh

On admission (day 9), her body temperature was 37.1 °C, blood pressure was 118/74 mmHg, heart rate was 106 beats per minute with a regular rhythm, respiratory rate was 16 breaths per minute, and peripheral oxygen saturation (SpO₂) was 98% on room air. She was alert and fully oriented. Physical examination revealed multiple, discrete, millet-sized palpable purpuras predominantly on the extremities, including the palms, without confluence or associated pain or pruritus (Figure 1).

No palpable superficial lymph nodes were detected. Cardiopulmonary and neurological examinations were unremarkable.

Laboratory tests showed a white blood cell (WBC) count of 6,700/μL and a mildly elevated C-reactive protein (CRP) level of 1.64 mg/dL. Liver enzymes were markedly elevated, with aspartate aminotransferase (AST) 108 IU/L, alanine aminotransferase (ALT) 125 IU/L, and lactate dehydrogenase (LDH) 546 IU/L. Platelet count was within normal limits. Coagulation studies were unremarkable, with a prothrombin time (PT) of 117.9%. Serological tests for hepatitis B surface antigen and hepatitis C antibody were negative. Epstein-Barr virus serology showed positive immunoglobulin G (IgG) but negative immunoglobulin M (IgM) antibodies, indicating past infection. Cytomegalovirus immunoglobulin (Ig) M was negative, and IgG was positive (Table 1).

**Table 1:** Initial laboratory data of the patient Reference ranges are based on the standards of our hospital’s clinical laboratory. Abbreviations: WBC, white blood cell; CRP, C-reactive protein; PT, prothrombin time; APTT, activated partial thromboplastin time; ANA, antinuclear antibody; AMA, antimitochondrial antibody; Cu, copper; HBs, hepatitis B surface; HCV, hepatitis C virus; CMV, cytomegalovirus; EB, Epstein–Barr virus; VCA, viral capsid antigen; EBNA, Epstein–Barr nuclear antigen; Ig, immunoglobulin; Na, sodium; K, potassium; Cl, chloride

Parameter	Level	Reference
White blood cells	6.7	3.5–9.1 × 10^3^/μL
Neutrophils	65.5	44.0–72.0%
Lymphocytes	25.7	18.0–59.0%
Hemoglobin	12.2	11.3–15.2 g/dL
Hematocrit	35.1	33.4–44.9%
Mean corpuscular volume	92.0	79.0–100.0 fl
Platelets	13.0	13.0–36.9 × 10^4^/μL
Total protein	6.1	6.5–8.3 g/dL
Albumin	3.2	3.8–5.3 g/dL
Total bilirubin	0.6	0.2–1.2 mg/dL
Aspartate aminotransferase	108	8–38 IU/L
Alanine aminotransferase	125	4–43 IU/L
Lactate dehydrogenase	546	121–245 U/L
Blood urea nitrogen	8.2	8–20 mg/dL
Creatinine	0.42	0.40–1.10 mg/dL
Serum Na	131	135–150 mEq/L
Serum K	3.3	3.5–5.3 mEq/L
Serum Cl	88	98–110 mEq/L
Ferritin	541	14.4–303.7 ng/mL
CRP	1.64	<0.30 mg/dL
PT	117.9	70-130 %
APTT	28.0	25.0-40.0 sec
IgG	1799	870–1700 mg/dL
IgM	99	35–220 mg/dL
IgA	307	110–410 mg/dL
ANA	40>	40>
AMA	Negative	Negative
Serum Cu	117	66-130 μg/dL
HBs antigen	0.01	0.00-0.04 IU/mL
HCV antibody	0.01	0-0.99 S/CO
CMV IgG	(+)	Negative
CMV IgM	Negative	Negative
EB VCA IgG	10.0	0-0.5
EB VCA IgM	10.8	0-0.5
EB EBNA IgG	2.2	0-0.5
Urine test	-	-
Leukocyte	Negative	Negative
Protein	Negative	Negative
Blood	Negative	Negative

**Figure 2:**
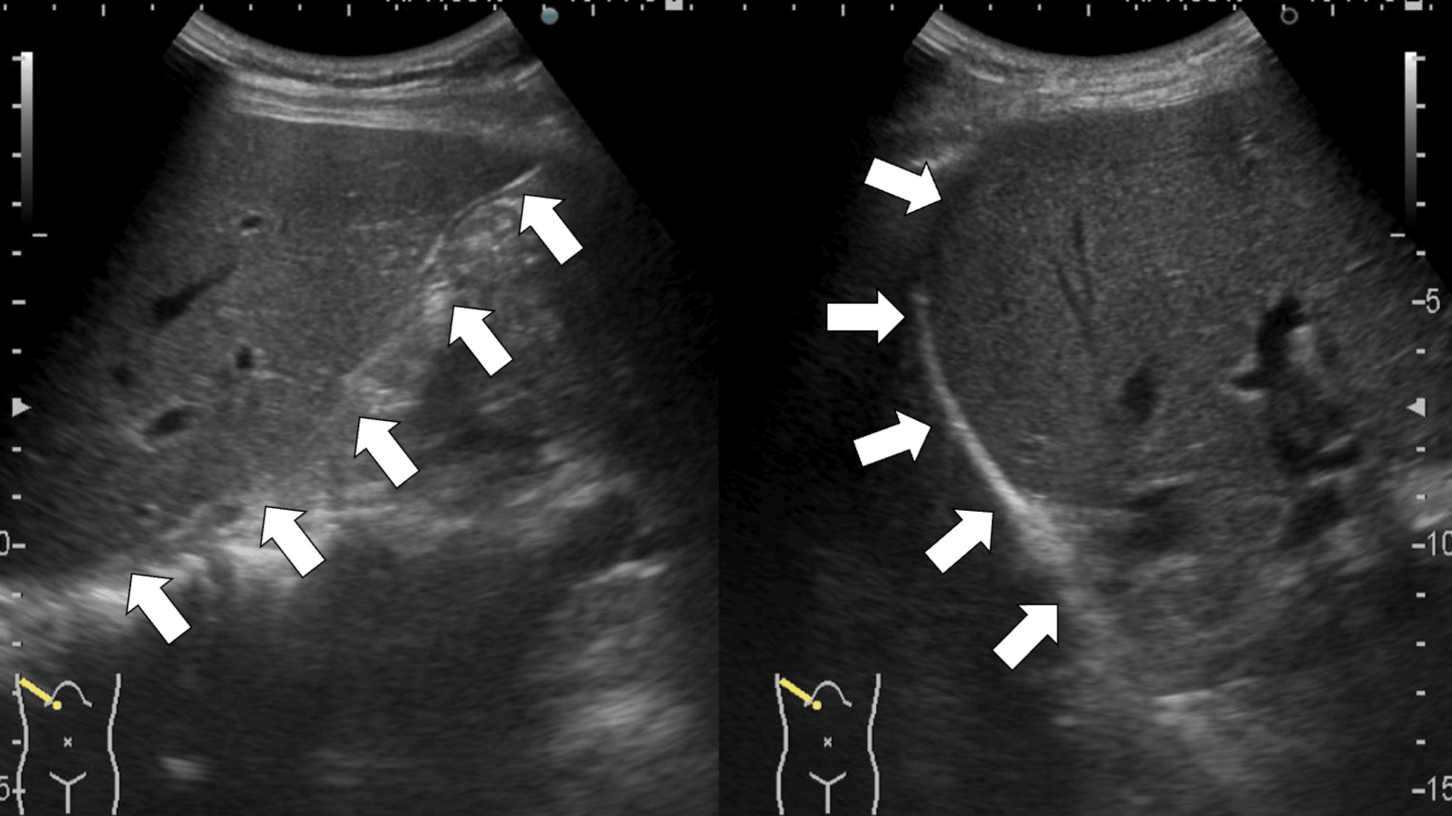
Abdominal ultrasound revealing no hepatomegaly (white arrows)

However, the acute-phase serology of immunoglobulin M and G, and the polymerase chain reaction test for *R. japonica *were negative on the day of admission. Abdominal ultrasonography revealed no hepatosplenomegaly (Figure 2).

**Figure 3:**
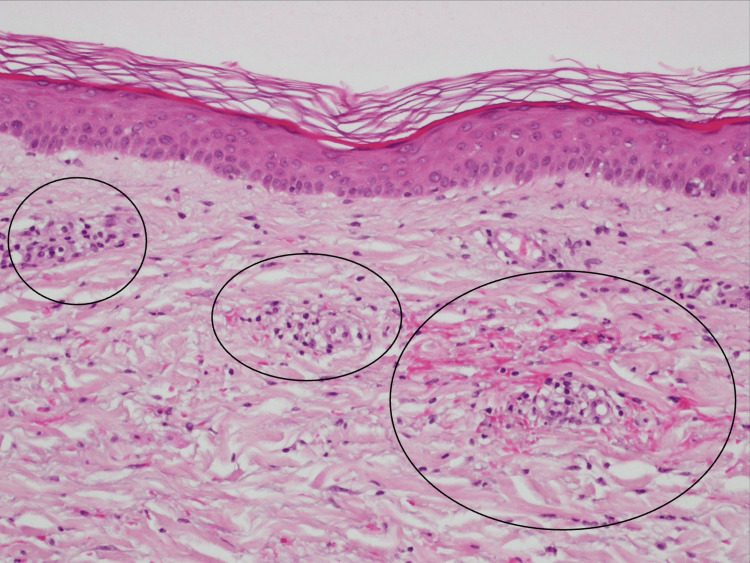
Histopathological examination of the right thigh’s erythema, revealing vasculitis with perivascular lymphohistiocytic infiltration and endothelial swelling (black circles)

Given her fever, diffuse rash, laboratory findings, and history of outdoor exposure in an endemic area of JSF, JSF was clinically suspected after evaluating other differentials such as drug eruption, viral exanthema, and other rickettsioses (e.g., scrub typhus). Empirical therapy with oral minocycline 200 mg/day was initiated immediately upon admission. On hospital day 2 (symptom day 11), a skin biopsy was obtained from an erythematous lesion on the lateral part of the right thigh. Histopathological examination revealed vasculitis with perivascular lymphohistiocytic infiltration and endothelial swelling, findings suggestive of rickettsial infection, considering the clinical course (Figure 3).

Her body temperature normalized by hospital day 2, and liver enzyme levels gradually improved with supportive care, including hydration and antimicrobial therapy. Facial edema subsided progressively, and the rash faded over the subsequent days. Her arthralgia and anorexia also resolved, and she was discharged on hospital day 10 (symptom day 19). The convalescent-phase serum obtained 14 days after discharge (symptom day 33) demonstrated a tenfold increase in immunoglobulin G titers for Rickettsia japonica, confirming the diagnosis of JSF. At the follow-up visit 14 days post-discharge, all cutaneous lesions had completely resolved, including the erythematous lesion on the right thigh.

## Discussion

This case emphasizes the crucial role of clinical judgment in the early management of suspected JSF, particularly when diagnostic serological tests are negative during the acute phase. The patient exhibited hallmark features suggestive of JSF: high fever, generalized erythematous rash with involvement of the palms and soles, arthralgia, facial edema, and hepatic dysfunction. These findings, combined with her epidemiological background of regular agricultural activity in a rural area endemic for *R. japonica*, strongly supported the clinical suspicion of a rickettsial etiology [5-7]. Notably, the absence of an eschar and the initial negative serology posed diagnostic challenges. However, the empirical initiation of minocycline led to rapid defervescence, gradual normalization of liver enzyme levels, and complete clinical recovery. Serological confirmation was eventually obtained in the convalescent phase, demonstrating a tenfold increase in antibody titers.

Rickettsial diseases, including JSF and scrub typhus, are caused by obligate intracellular bacteria that can lead to life-threatening complications if left untreated [8,9]. Early recognition and timely treatment are critical. Previous studies have shown that delays in initiating appropriate antibiotic therapy are associated with increased morbidity and mortality, especially in elderly patients and those with multi-organ involvement [10,11]. This case illustrates that empirical antibiotic therapy--primarily with doxycycline or minocycline--should be strongly considered when clinical and epidemiological features are highly suggestive, even in the absence of confirmatory laboratory evidence. Clinicians must remain vigilant in such situations, as early intervention can be life-saving.

In addition to the clinical response, histopathological examination of a skin lesion provided crucial diagnostic support. The biopsy revealed perivascular lymphohistiocytic infiltration and endothelial cell swelling, consistent with small-vessel vasculitis, which is a histological hallmark of rickettsial infections [12,13]. *R. japonica* invades vascular endothelial cells, leading to direct endothelial damage, inflammation, and systemic vasculitic manifestations [14]. In diagnostically ambiguous cases, skin biopsy findings can offer valuable insights, helping distinguish rickettsial infections from viral exanthems, drug eruptions, or autoimmune conditions [15]. Notably, while other causes of small-vessel vasculitides, such as leukocytoclastic vasculitides, antineutrophil cytoplasmic antibodies (ANCA)-associated vasculitides, or connective tissue disease-related vasculitides, may show similar perivascular inflammatory patterns, these typically feature prominent neutrophilic infiltration, fibrinoid necrosis, or immune-complex deposition, which were not observed in this case [15]. In contrast, the lymphohistiocytic infiltration with endothelial swelling seen here is characteristic of rickettsial vasculitis. While not always performed routinely, skin biopsies should be considered in patients with compatible cutaneous findings when serology is inconclusive or delayed [16]. In this case, the histological evidence further reinforced the clinical diagnosis and guided appropriate management.

## Conclusions

This case underscores the importance of the early clinical diagnosis and empirical treatment of suspected JSF, particularly in elderly patients living in endemic rural areas. Even in the absence of an eschar and with negative acute-phase serology, characteristic systemic symptoms, epidemiological risk factors, and supportive histopathological findings can justify prompt initiation of therapy. Early recognition and timely administration of tetracyclines can lead to rapid recovery and prevent potentially severe complications. Integrating clinical, epidemiological, and pathological data remains essential for the timely and effective management of JSF.
